# Terahertz Cross-Correlation Spectroscopy and Imaging of Large-Area Graphene

**DOI:** 10.3390/s23063297

**Published:** 2023-03-21

**Authors:** Bjørn Hübschmann Mølvig, Thorsten Bæk, Jie Ji, Peter Bøggild, Simon Jappe Lange, Peter Uhd Jepsen

**Affiliations:** 1 Department of Electrical and Photonics Engineering, Technical University of Denmark, 2800 Kongens Lyngby, Denmark; thbak@fotonik.dtu.dk (T.B.); slla@dtu.dk (S.J.L.); 2 GLAZE Technologies Aps, 2950 Vedbæk, Denmark; 3 Department of Physics, Technical University of Denmark, 2800 Kongens Lyngby, Denmark; jieji@dtu.dk (J.J.); pbog@dtu.dk (P.B.)

**Keywords:** THz time-domain spectroscopy, THz cross-correlation spectroscopy, THz quasi-time-domain spectroscopy, graphene

## Abstract

We demonstrate the use of a novel, integrated THz system to obtain time-domain signals for spectroscopy in the 0.1–1.4 THz range. The system employs THz generation in a photomixing antenna excited by a broadband amplified spontaneous emission (ASE) light source and THz detection with a photoconductive antenna by coherent cross-correlation sampling. We benchmark the performance of our system against a state-of-the-art femtosecond-based THz time-domain spectroscopy system in terms of mapping and imaging of the sheet conductivity of large-area graphene grown by chemical vapor deposition (CVD) and transferred to a PET polymer substrate. We propose to integrate the algorithm for the extraction of the sheet conductivity with the data acquisition, thereby enabling true in-line monitoring capability of the system for integration in graphene production facilities.

## 1. Introduction

Since graphene was first made available by mechanical exfoliation of graphite [1], this two-dimensional material has continued to attract a significant amount of attention due to its remarkable mechanical, thermal, optical, and electronic properties [2,3]. It is predicted to have applications in a vast number of areas, including electronics, photonics, optoelectronics, and transistor- and composite-material manufacturing [4,5,6,7], and graphene material and device manufacturing continues to expand and thus creates a stronger need for reliable, fast, and non-destructive quality inspection techniques [8].

Terahertz time-domain spectroscopy (THz-TDS) has been demonstrated to be a versatile technique for the non-destructive retrieval of the electrical properties of graphene [9,10,11,12]. While commercial THz-TDS devices are capable of providing both accurate and precise measurements, they are not easily implemented in production facilities, while THz systems aimed at high-speed commercial applications do not provide the deep level of information needed to establish key performance characteristics of graphene films. Other techniques for the retrieval of the electrical properties of graphene include microwave impedance microscopy and micro four-point probe measurements, where the former, such as THz-TDS, is a non-contact technique, and the latter requires direct physical contact with the sample [13].

THz-TDS was first introduced in 1989 through the utilization of photoconductive antennas (PCAs) and femtosecond (fs) lasers [14]. In a typical setup, a PCA emitter is optically excited by the laser pulse, which, through charge carrier acceleration, emits THz radiation that subsequently is coherently detected via photoconductive sampling in a PCA detector [15]. Other methods for THz generation and detection include optical rectification in nonlinear crystals and third-order nonlinear effects in air plasmas, both methods utilizing fs lasers as well [16,17].

Instead of generating THz radiation using pulsed lasers, it was proposed in 1999 to excite PCAs with a temporally incoherent light source and measure the cross-correlation between the emitted incoherent radiation and the incoherent pump light [18,19]. Similar systems have since been fabricated employing different temporally incoherent light sources such as superluminescent diodes or multi-mode laser diodes [20,21,22,23]. In this work, we present a novel THz cross-correlation spectroscopy (CCS) device capable of obtaining results similar to commercial systems, while being smaller, easier to operate, and less complex, which allows for significantly cheaper production. We have previously demonstrated CCS applications of thickness measurements based on time-of-flight algorithms [24,25], and here we focus on the spectroscopic capabilities of the CCS system in sensing applications.

## 2. THz Cross-Correlation Spectroscopy

**Figure 1:**
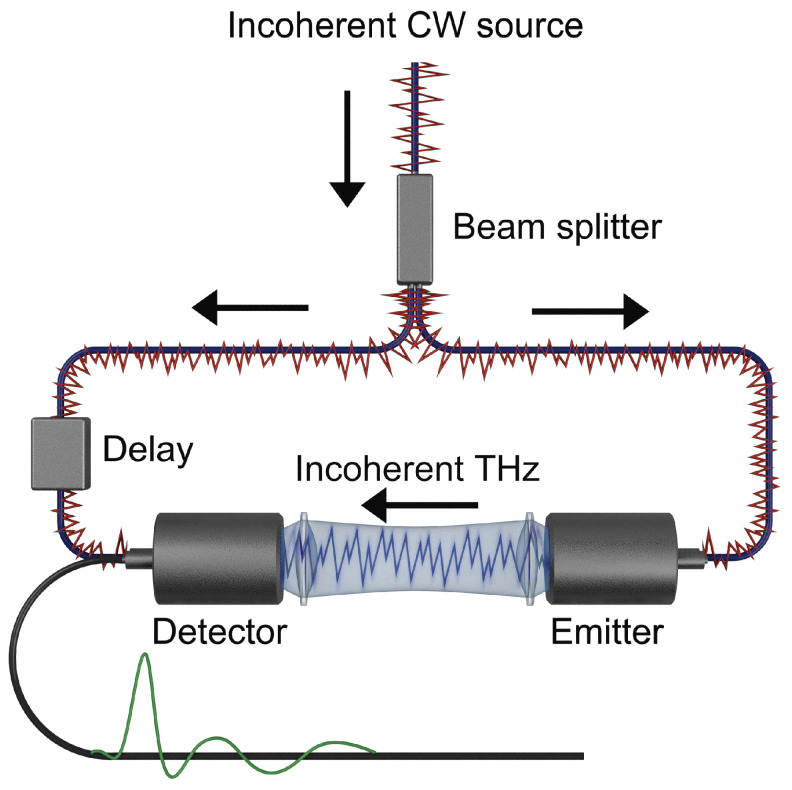
A THz-CCS experiment utilizing PCAs and a temporally incoherent light source for detection and emission. The green trace on the lower black line indicates a typical electrical field profile as a function of time, extracted from the THz-CCS measurements.

The main components of a typical CCS setup using PCAs is shown schematically in Figure 1. A light source source with low temporal coherence generates an intensity $I\left(t\right)\propto {\left|E\left(t\right)\right|}^{2}$ , which is split into an emitter and a detector arm, respectively. In the emitter arm, a biased PCA is illuminated, which generates THz radiation $E_{\mathrm{THz}}\left(t\right)$ with a similar low temporal coherence as the light source. Note that at any two points of equal optical distance from the beamsplitter, the incoherent light intensities in the two arms are identical.

Both emitter and detector operate by the standard principles of THz-frequency photomixing [26]. Assuming a spatially uniform intensity profile and ignoring antenna effects, the emitted THz field is proportional to the temporal derivative of the photocurrent induced by free carriers generated by the incoherent light. The photocurrent is the product of the photoconductivity $\sigma_{e}\left(t\right)$ and the constant emitter bias voltage $U_{e}$ . Taking the impulse response $G_{e}\left(t\right)$ of the emitter into account, we find that

(1) $$\begin{array}{c} E_{\mathrm{THz}}\left(t\right)\propto \frac{d}{dt}\sigma_{e}\left(t\right)\propto \frac{d}{dt}\int_{-\infty }^{t}G_{e}\left(t^{\prime }\right)I\left(t-t^{\prime }\right)dt^{\prime }. \end{array}$$

In the detector, as in the emitter, the incoherent light generates free carriers inducing photoconductance $\sigma_{d}\left(t\right)$ ,

(2) $$\begin{array}{c} \sigma_{d}\left(t\right)\propto \int_{-\infty }^{t}G_{d}\left(t^{\prime }\right)I\left(t-t^{\prime }\right)dt^{\prime }, \end{array}$$

where $G_{d}\left(t\right)$ is the impulse response of the detector antenna, and the incoming THz field drives a photocurrent J(t) . Effectively, when integrating using, e.g., lock-in detection over a time period much greater than any characteristic time in the system, a current $J_{\mathrm{CC}}\left(t\right)$ proportional to the cross-correlation

(3) $$J_{CC}\left(t\right)\propto \int_{-\infty }^{\infty }E_{THz\;}\left(t^{\prime }\right)\sigma_{d}\left(t^{\prime }-t\right)dt^{\prime }.$$

between $E_{\mathrm{THz}}\left(t\right)$ and σ(t) is measured. Inserting Equations (1) and (2) in Equation (3) and applying the Fourier transform, we find that

(4) $${\tilde{J}}_{CC}\left(\omega \right)\propto \omega {\tilde{G}}_{e}\left(\omega \right){\tilde{G_{d}}}^{*}\left(\omega \right){\left|\tilde{I}\left(\omega \right)\right|}^{2},$$

which is a coherent spectrum independent of the phase of the light source. Despite the use of an incoherent light source, the fact that the cross-correlation depends on the absolute square of the intensity $\tilde{I}\left(\omega \right)$ means that the measured cross-correlation has a pulse-like shape, when transformed back to the time domain. By properly choosing the bandwidth of the light source, the intensity $I\left(t\right)\propto {\left|E\left(t\right)\right|}^{2}$ of it will contain frequency-mixed terms in the THz range.

## 3. Extraction of Sheet Conductivity of Thin Films in the THz Range

Upon insertion of a sample between the emitter and the detector, the field is modified. The interaction is described in the frequency domain by a transfer function $\tilde{H}\left(\omega \right)$ , which modifies the measured photocurrent to

(5) $$\begin{array}{c} \tilde{J_{s}}\left(\omega \right)={\tilde{J}}_{\mathrm{CC}}\left(\omega \right)\tilde{H}\left(\omega \right). \end{array}$$

**Figure 2:**
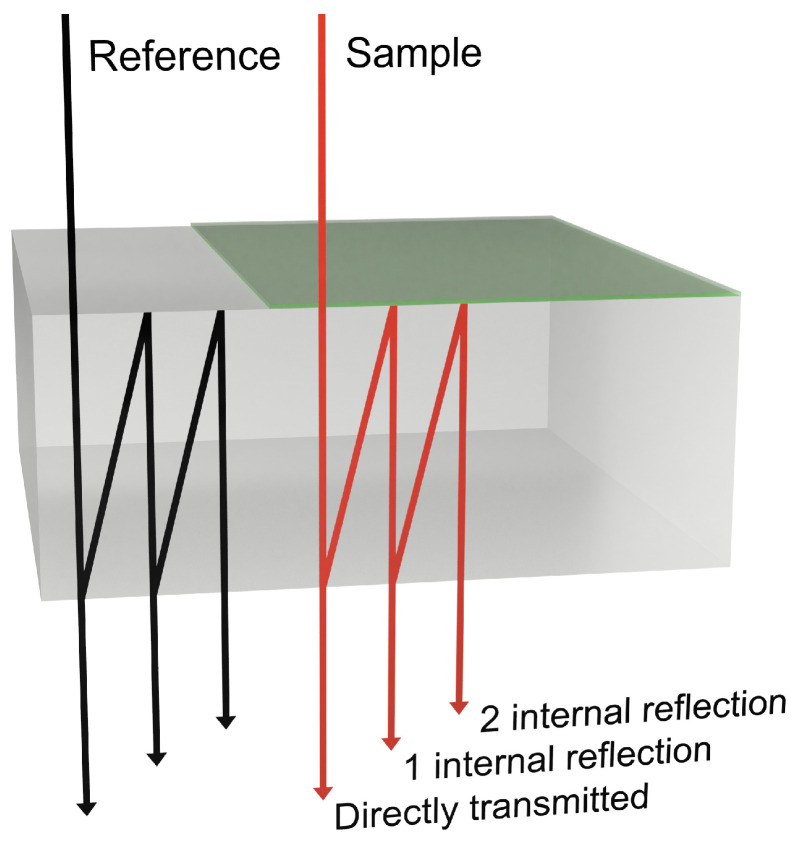
Sketch of different contributions to the detected THz signal from multiple internal reflections. The gray box indicates the substrate and the green sheet the sample, such as graphene.

The transfer function is set up by analyzing how plane waves of light propagate through the sample in a transmission experiment as displayed in Figure 2. Especially in situations where the sample is placed on a thin, low-index substrate such as a polymer film, the inclusion of internal reflections becomes important for a quantitative analysis of the conductive properties of the thin film as the directly transmitted signals and the internal reflections may overlap in time [27].

The total transmitted field $E_{\mathrm{tot}}$ is the sum of the directly transmitted field and the fields having undergone *n* internal reflections, i.e.,

(6) $$\begin{array}{cc} {\tilde{E}}_{\mathrm{tot}} & ={\tilde{E}}_{0}\cdot t_{12}t_{23}e^{i\delta }\sum_{n=0}^{\infty }{\left(r_{23}r_{21}e^{i2\delta }\right)}^{n}\\ \; & ={\tilde{E}}_{0}\cdot \frac{t_{12}t_{23}e^{i\delta }}{1-r_{23}r_{21}e^{i2\delta }}={\tilde{E}}_{0}\left(\omega \right)\cdot \tilde{H}\left(\omega \right), \end{array}$$

where $r_{ij}$ and $t_{ij}$ are the standard Fresnel reflection and transmission coefficients for reflection/transmission between media *i* and *j* and $\delta =\frac{n_{2}\omega d}{c}$ is the acquired phase through medium 2 of thickness *d* and refractive index $n_{2}\left(\omega \right)$ .

When a thin film of a conducting material such as graphene is present on the interface between medium 1 and 2, the relevant transmission and reflection coefficients are described by the Tinkham equations [28] given by

(7) $$\begin{array}{cc} t_{12} & =\frac{2n_{1}}{n_{1}+n_{2}+Z_{0}\sigma \left(\omega \right)}, \end{array}$$

(8) $$\begin{array}{cc} r_{21} & =\frac{n_{1}-n_{2}-Z_{0}\sigma \left(\omega \right)}{n_{1}+n_{2}-1+Z_{0}\sigma \left(\omega \right)}, \end{array}$$

where $Z_{0}$ is the free-space impedance and σ(ω) is the complex Drude conductivity given by

(9) $$\begin{array}{c} \sigma \left(\omega \right)=\frac{\sigma_{\mathrm{DC}}}{1-\omega \tau }, \end{array}$$

where $\sigma_{\mathrm{DC}}$ and τ are the material-specific DC conductivity and momentum relaxation time, respectively.

To extract the conductivity experimentally, a sample measurement $E_{\mathrm{sample}}$ of graphene on a substrate and a reference measurement $E_{\mathrm{ref}}$ of the substrate are taken. When divided, $\sigma_{\mathrm{DC}}$ and τ can be extracted by fitting the resulting expression

(10) $$\begin{array}{c} \frac{{\tilde{E}}_{\mathrm{sample}}}{{\tilde{E}}_{\mathrm{ref}}}=\frac{t_{af}\left(1-r_{sa}r_{sa}e^{i2\delta }\right)}{t_{as}\left(1-r_{sa}r_{fa}e^{i2\delta }\right)}, \end{array}$$

where subscripts *s, a*, and *f* refer to substrate, air, and film, respectively, using either deterministic or stochastic fitting algorithms such as basin hopping or differential evolution [29,30].

## 4. Experimental Setup

**Figure 3:**
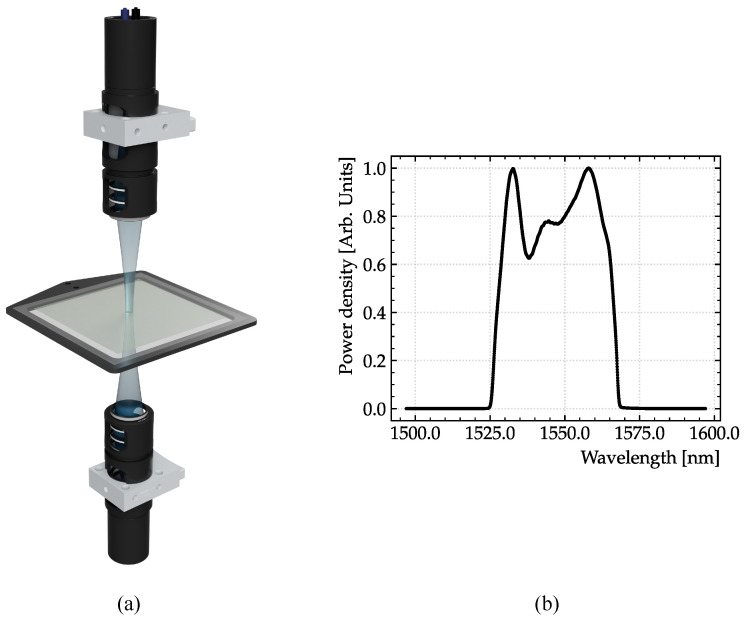
(**a**) Illustration of the transmission geometry. The THz light is focused and transmitted through a sample of graphene on a dielectric substrate. (**b**) C-band ASE output power density.

Two transmission mode experiments on the same sample of graphene at normal angle of incidence have been executed. The experimental setup is shown in Figure 3a. The first experiment is performed with a commercial THz-TDS system (TOPTICA Teraflash Smart) and serves as a high-precision benchmark. The second experiment is conducted with our THz CCS device. A fiber-coupled C-band amplified spontaneous emission (ASE) light source is split into two arms. As displayed in Figure 3b, the ASE output covers the range from 1525 to 1565 nm, which allows for the generation of photomixed frequencies of up to 5 THz. Due to the low-pass characteristics of the emitter and detector PCAs, bandwidths higher than 2 THz are not currently obtainable with the used component. Each beam is sent through one of the two fiber stretchers, which together are capable of creating a delay of 100 picoseconds (ps), before being coupled to an InGaAs-based emitter antenna and an InGaAs-based detector antenna, respectively (TOPTICA InGaAs Photomixers). The cross-correlation is measured via lock-in detection.

**Figure 4:**
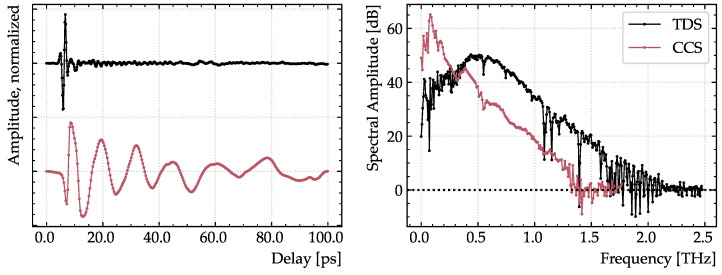
Traces (**left**) and spectra (**right**) of the commercial THz-TDS system (black) and the novel THz-CCS device (red). The TDS trace has been offset. Key metrics are summarized in Table 1.

Key metrics for the used devices as operated are summarized in Table 1. The TDS device has a bandwidth of 2.3 THz compared to 1.4 THz and a dynamic range of 50 dB compared to 58 dB of the CCS device at acquisition times of 3 s for the TDS device and 30 s for the CCS device. Due to the difference in acquisition time, the metrics cannot be directly compared—a relatively low acquisition time for the TDS device was used since it resulted in satisfactory results. In Figure 4, example time traces (left) and spectra (right) of both devices are shown. A Tukey window function with a taper length of 5 ps has been applied to both traces. The CCS trace does not converge to zero in the end of the time window, which influences the spectral analysis.

**Figure 5:**
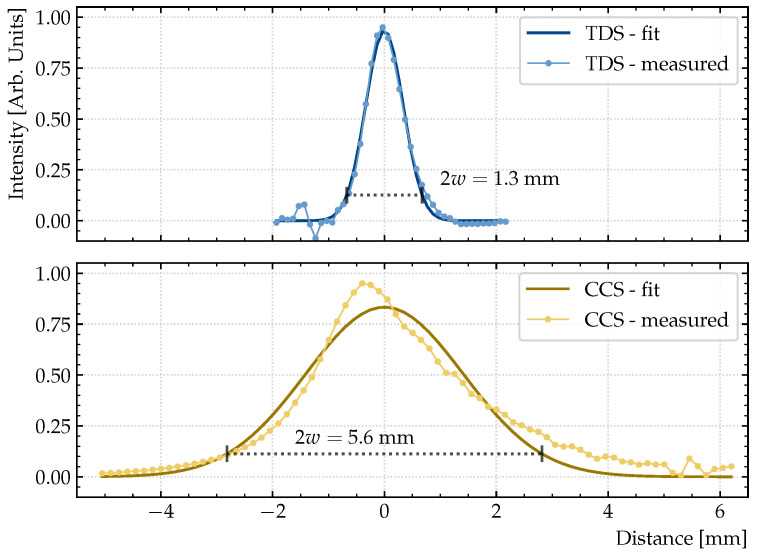
Knife edge scans of the TDS device (blue) and the CCS device (yellow) with Gaussian fits to determine beam spot sizes. Black dotted lines indicate the spot sizes, which are found to be 1.3 mm for the TDS device and 5.6 mm for the CCS device.

Additionally, the spot sizes were determined through knife-edge scans in ambient air, shown in Figure 5. The profiles were extracted from the series of full time-domain traces $E_{\mathrm{THz}}\left(x\right)$ as a function of the knife edge position *x* by calculating the energy contained in each trace proportional to $U\left(x\right)\propto \int E^{2}\left(t\right)dt$ . The beam intensity profile was then found by the derivative, $I_{\mathrm{THz}}\left(x\right)=dU/dx$ .

The spot sizes are found to be 1.3 mm for the TDS device and 5.6 mm for the CCS device, where the spot size is defined as twice the width extracted from fitting the beam intensity profile with a Gaussian function of the form

(11) $$\begin{array}{c} I_{\mathrm{THz}}\left(x\right)=A\exp\left(-\frac{2x^{2}}{w^{2}}\right), \end{array}$$

where *A* is a normalization constant, *x* is the spatial position, and *w* is the beam width.

## 5. Sample Fabrication

**Figure 6:**
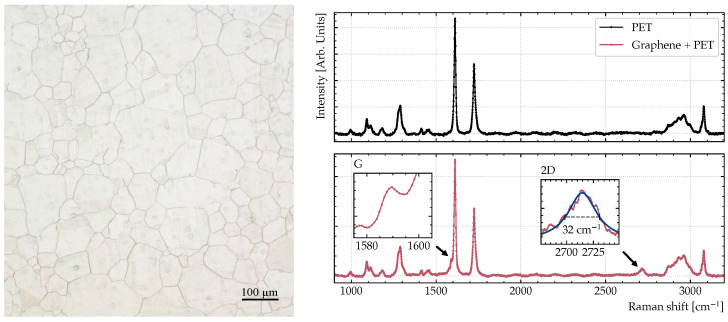
Optical microscopy image (**left**) and Raman spectra (**right**) of the PET substrate and the graphene sample on top. The annotated peaks of the G and 2D bands of graphene are consistent with the presence of bi- or trilayer graphene.

The graphene sample is grown by chemical vapor deposition (CVD) on copper foil and then transferred to a transparent PET substrate with standard wet transfer techniques [31]. During the transfer process, a polymer film model from a 1.3 wt.% solution of 2200 K Poly(methyl methacrylate) (PMMA) is used as a support. The sample is then placed in acetone for 1 h to remove the PMMA and finally cleaned by isopropanol. Figure 6 shows an optical microscopy image (left) and the Raman spectra of the PET substrate and the graphene on top of the PET substrate (right). The peaks of the G and 2D bands located at 1588 ${\mathrm{cm}}^{-1}$ and 2720 ${\mathrm{cm}}^{-1}$ in the spectra are the most prominent features in the Raman spectra of graphene, which confirms the presence of graphene in the sample [32]. The 2D band has a Lorentzian full width at half maximum of 32 ${\mathrm{cm}}^{-1}$ , which is consistent with a sample of 2–3 layers of graphene [33,34].

**Figure 7:**
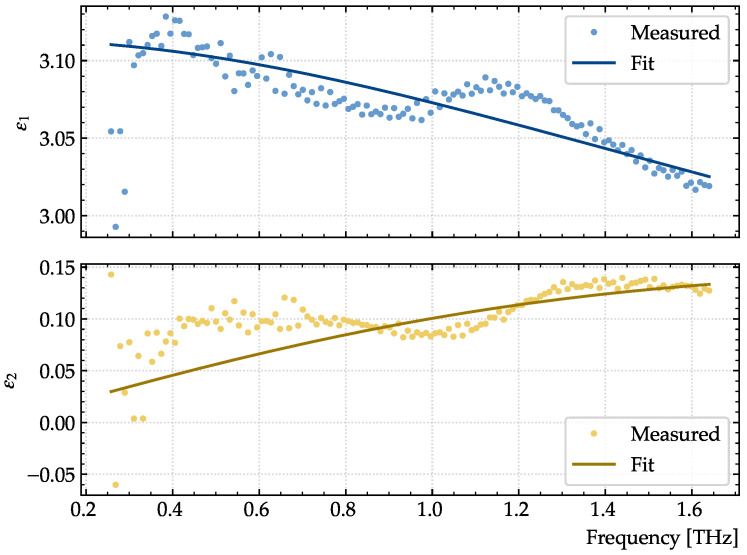
Extracted real (top) and imaginary (bottom) parts of the dielectric permittivity of the PET substrate supporting the graphene films. The fitted values are given by a Debye model with parameters $\epsilon_{\infty }=2.91$ , $\epsilon_{s}=3.21$ , and τ=65.9 fs.

For better extraction of the graphene conductivity parameters, the thickness and dielectric permittivity of the substrate have been characterized experimentally with the TDS device and determined to be 208 μ m thick and well described ( $R^{2}=96\%$ ) by a Debye model given by

(12) $$\begin{array}{c} \epsilon \left(\omega \right)=\epsilon_{\infty }+\frac{\epsilon_{s}-\epsilon_{\infty }}{1-i\omega \tau } \end{array}$$

with $\epsilon_{\infty }=2.91$ , $\epsilon_{s}=3.21$ and τ=65.9 fs. Many polymers display similar behavior in the low THz range, including a slightly decreasing real part of the permittivity and an increasing absorption towards higher frequencies [35]. The real and imaginary parts of the extracted dielectric permittivity are shown in Figure 7. The oscillatory feature at 0.8–1.3 THz was not observed in previous measurements [36], and we attribute it to an incomplete cancellation of multiple reflections during the data extraction.

The Debye model describes the relaxation processes and displays the same qualitative behavior of the dielectric function. Hence, it is frequently used to model the dielectric properties of polymers in this range [37,38].

## 6. Experimental Results

**Figure 8:**
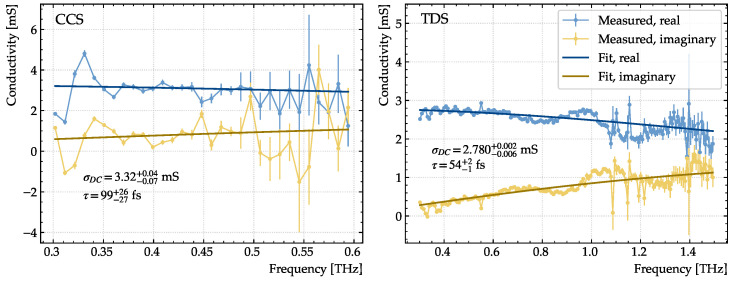
Examples of real (blue) and imaginary (yellow) parts of the conductivity determined with the CCS system (**left**) and the TDS system (**right**) through fitting Equation (10) with a stochastic optimization algorithm.

A 9 cm × 9 cm sheet of graphene was raster-scanned in ambient air in a serpentine pattern with a stepsize of 3 mm, corresponding to 961 single-pixel measurements. For each pixel, the Drude conductivity was extracted by fitting with Equation (10) using the stochastic optimization algorithm differential evolution [39]. An example of the extracted conductivity using each device is shown in Figure 8, where errors denote 68% confidence bounds. The conductivity spectra shown in Figure 8 are not recorded at the same position on the sample, and hence the absolute values cannot be directly compared.

Since the TDS system has a small spot size compared to the pixel dimensions, >99.99% of the THz radiation is contained within each pixel, assuming a Gaussian beam profile. For the CCS device with a much larger spot size, only 35.6% of the radiation is contained, leading the extracted conductivities to be a weighted mean of the pixel in question and its neighbors. To provide a reasonable basis for comparison, the pixel values measured using the TDS device are therefore convolved with a Gaussian filter with a width equal to the beam width of the CCS device.

**Figure 9:**
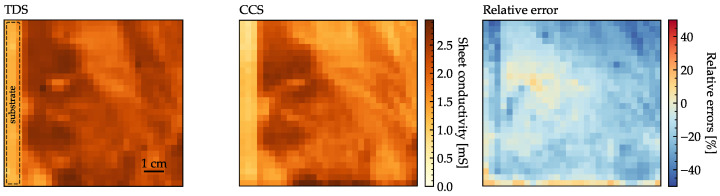
Measured conductivity of a 9 cm × 9 cm sheet of graphene using THz-TDS (**left**), THz-CCS (**center**) and the relative error between them (**right**). The mean of the errors is −16.3% , and the standard deviation is 10.6% .

The spatial maps of the measured conductivities using both devices are shown in Figure 9. The CCS measurements display the same conductivity pattern of spotted areas of higher and lower conductivity as the TDS measurements, and the measured DC conductivities have a linear correlation of 84.5% . The left-most vertical line corresponds to an area of the substrate without graphene, as indicated on the TDS conductivity plot. The CCS device is more biased towards measuring lower conductivities than the TDS device, and the bias displays spatial correlations as seen in the right panel of Figure 9. Specifically, the mean error is −16.3% and the standard deviation on the relative errors given by

(13) $$\begin{array}{c} \sigma_{err}=\mathrm{std}\left(\frac{\sigma_{\mathrm{DC}}^{CCS}-\sigma_{\mathrm{DC}}^{TDS}}{\sigma_{\mathrm{DC}}^{TDS}}\right) \end{array}$$

is 10.6% . The reason for the bias is discussed in the next section.

**Figure 10:**
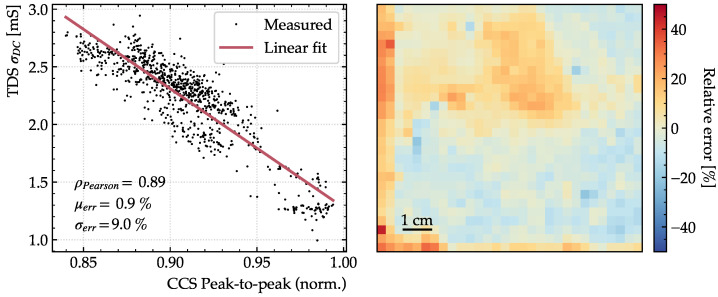
$\sigma_{\mathrm{DC}}$ measured using TDS against peak-to-peak measurements using CCS along with a linear fit (**left**) and the spatial relative error, when the peak-to-peak is used to predict $\sigma_{\mathrm{DC}}$ (**right**).

Since the transmission decreases when the DC conductivity increases, the peak-to-peak value is correlated with the DC conductivity. In Figure 10, the DC conductivity determined with TDS is shown to display a strong linear correlation with the peak-to-peak values of the CCS measurements with a linear correlation of 89%. If the peak-to-peak values are used to predict $\sigma_{\mathrm{DC}}$ with a linear model (red) instead of retrieving it with a fitting procedure, the mean error is reduced to 0.9% and the standard deviation of the relative errors given by Equation (13) is reduced to 9.0% . Similarly to the relative errors on the DC conductivities extracted with the fitting procedure shown in Figure 9, the errors on the DC conductivities extracted with peak-to-peak procedure display spatial correlations as shown by coherent areas of positive or negative relative error.

## 7. Discussion of Results

The CCS device is capable of measuring the DC conductivity of graphene with a standard deviation on the error distribution of 10.6% compared to the TDS benchmark. As shown in the previous section, patterns of high and low conductivity areas are captured and match the areas measured with the TDS device. However, as previously mentioned, the DC conductivities extracted with the fitting procedure using the CCS device are biased towards lower values. This bias is believed to primarily stem from drift in the optical path length in the fiber stretchers, where the length of the true time window increases over longer periods of operation, which is not reflected on the x-axis.

We believe that correctly compensating for the drift can remove the bias. This is currently being explored.

**Figure 11:**
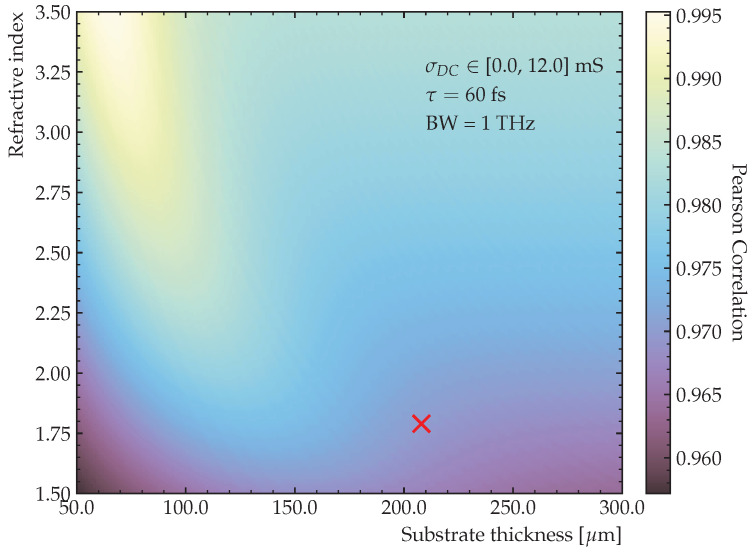
Linear correlation between the peak-to-peak value and the DC conductivity in the range [0.0,12.0] mS for a simulated 1 THz bandwidth pulse for different substrate thicknesses and refractive indices. The red cross denotes a parameter set close to the used experimental settings.

In the previous section, we demonstrated how the DC conductivity can be determined with higher precision from the peak-to-peak value of the trace. In Figure 11, the linear correlation between the peak-to-peak value and the DC conductivity has been simulated for an identical experimental setup. For each value of the substrate thickness and refractive index, the corresponding pairs of $\sigma_{\mathrm{DC}}$ and peak-to-peak values are calculated for a simulated pulse with a bandwidth of 1 THz, τ=60fs , and $\sigma_{\mathrm{DC}}$ , linearly varying between 0 and 12 mS. Each pixel value is then computed as the linear correlation between the peak-to-peak and $\sigma_{\mathrm{DC}}$ values, showing that it is possible to achieve correlations higher than 99%. The red cross denotes the point ρ(d=208μm,n=1.79)=97.0% , which is close to the settings at which the experiments presented here have been conducted.

Extracting $\sigma_{\mathrm{DC}}$ from the peak-to-peak values removes the need to acquire the entire trace since the peak-to-peak value can be determined using only a fraction of the trace points. Since each CCS-trace consists of 200 data points, the acquisition time can ideally be reduced by up to a factor of 100 with a very moderate increase in measurement error merely by only scanning the maximum and minimum of the trace. Furthermore, it removes the error related to the signal not settling to zero within the limited time window and other issues with fit-based extraction procedures. At such rates, inline conductivity measurements of graphene become not only possible but viable.

## 8. Conclusions

We have demonstrated that our novel THz-CCS device is capable of measuring the electrical properties of graphene. The measurements have been compared to what is achievable with a state-of-the-art commercial THz-TDS device, resulting in a standard deviation on the relative errors of 9.0% . Combined with a modern, industry-oriented system design, THz-CCS devices are becoming an attractive option for inline, non-contact, high throughput, high-precision quality assurance for 2D semiconductor materials.

## Figures and Tables

**Table 1:** Summarization of key in situ properties used in the experiments for the THz-TDS and THz-CCS devices. Since different acquisition times are used, the performance of the devices cannot be compared directly.

	Bandwidth	Dynamic Range	Delay	Spot Size	Acq. Time
TDS	2.3 THz	50 dB @ 500 GHz	130 ps	1.3 mm	3 s
CCS	1.4 THz	58 dB @ 100 GHz	100 ps	5.6 mm	30 s

## Data Availability

Data underlying the results presented in this paper are not publicly available at this time but may be obtained from the authors upon reasonable request.

## Abbreviations

The following abbreviations are used in this manuscript:

THz-CCS	Terahertz cross-correlation spectroscopy
THz-TDS	Terahertz time domain spectroscopy
ASE	Amplified spontaneous emission
PCA	Photoconductive antenna
CVD	Chemical vapor deposition
PMMA	Poly(methyl methacrylate)
