# Correction: Mølvig et al. Terahertz Cross-Correlation Spectroscopy and Imaging of Large-Area Graphene. *Sensors* 2023, *23*, 3297

**DOI:** 10.3390/s25133895

**Published:** 2025-06-23

**Authors:** Bjørn Hübschmann Mølvig, Thorsten Bæk, Jie Ji, Peter Bøggild, Simon Jappe Lange, Peter Uhd Jepsen

**Affiliations:** 1 Department of Electrical and Photonics Engineering, Technical University of Denmark, 2800 Kongens Lyngby, Denmark; 2 GLAZE Technologies Aps, 2950 Vedbæk, Denmark; 3 Department of Physics, Technical University of Denmark, 2800 Kongens Lyngby, Denmark

In the original publication [1], there were two typographical errors in Equations (3) and (4) as published. The corrected Equations (3) and (4) appear below.

(3) $$J_{CC}\left(t\right)\propto \int_{-\infty }^{\infty }E_{THz\;}\left(t^{\prime }\right)\sigma_{d}\left(t^{\prime }-t\right)dt^{\prime }.$$

(4) $${\tilde{J}}_{CC}\left(\omega \right)\propto \omega {\tilde{G}}_{e}\left(\omega \right){\tilde{G_{d}}}^{*}\left(\omega \right){\left|\tilde{I}\left(\omega \right)\right|}^{2},$$

The authors state that the scientific conclusions are unaffected. This correction was approved by the Academic Editor. The original publication has also been updated.
